# A Nationwide Population-Based Study on the Survival of Patients with Pancreatic Neuroendocrine Tumors in The Netherlands

**DOI:** 10.1007/s00268-017-4278-y

**Published:** 2017-10-10

**Authors:** C. G. Genc, H. J. Klümpen, M. G. H. van Oijen, C. H. J. van Eijck, E. J. M. Nieveen van Dijkum

**Affiliations:** 1 Department of Surgery, Academic Medical Center, Meibergdreef 9, PO Box 22660, 1105 AZ Amsterdam, The Netherlands; 2 Department of Medical Oncology, Academic Medical Center, Amsterdam, The Netherlands; 3 Cancer Center Amsterdam, Amsterdam, The Netherlands; 4 Department of Research, Comprehensive Cancer Centers Netherlands (IKNL), Utrecht, The Netherlands; 5 Department of Surgery, Erasmus Medical Center, Rotterdam, The Netherlands

## Abstract

**Background:**

Large population-based studies give insight into the prognosis and treatment outcomes of patients with pancreatic neuroendocrine tumors (pNETs). Therefore, we provide an overview of the treatment and related survival of pNET in the Netherlands.

**Methods:**

Patients diagnosed with pNET between 2008 and 2013 from the Netherlands Cancer Registry were included. Patient, tumors and treatment characteristics were reported. Survival analyses with log-rank testing were performed to compare survival.

**Results:**

In total, 611 patients were included. Median follow-up was 25.7 months, and all-cause mortality was 42%. Higher tumor grade and TNM stage were significantly associated with worse survival in both the overall and metastasized population. The effect of distant metastases on survival was more significant in lower tumor stages (T1–3 *p* < 0.05, T4 *p* = 0.074). Resection of the primary tumor was performed in 255 (42%) patients. Patients who underwent surgery had the highest 5-year survival (86%) compared to PRRT (33%), chemotherapy (21%), targeted therapy and somatostatin analogs (24%) (all *p* < 0.001). Patients with T1M0 tumors (*n* = 115) showed favorable survival after surgical resection (*N* = 95) compared to no therapy (*N* = 20, *p* = 0.008). Resection also improved survival significantly in patients with metastases compared to other treatments (all *p* > 0.05). Without surgery, PRRT showed the best survival curves in patients with distant metastases. Grade 3 tumors and surgical resection were independently associated with survival (HR 7.23 and 0.12, respectively).

**Conclusion:**

Surgical resection shows favorable outcome for all pNET tumors, including indolent tumors and tumors with distant metastases. Prospective trials should be initiated to confirm these results.

## Introduction

According to Surveillance, Epidemiology and End Results (SEER) data, the incidence of neuroendocrine tumors (NET) showed a 2.7-fold increase between 1973 and 2004 [1]. The incidence of pancreatic NET (pNET) is estimated at 2/100.000, with a predicted rise faster than other malignant neoplasms [2]. Although pNET in general is considered to be indolent, some subtypes can be highly malignant and resistant to therapy [3]. As the majority of tumors are not associated with secretion of hormones that cause clinical symptoms, patients are predominantly diagnosed with disseminated disease for whom curation is not possible [4, 5]. SEER analyses demonstrate that 64% of patients with well-differentiated (G1 and G2) pNET are diagnosed with distant metastases and have a poor 5-year survival of 27%. For these patients, different treatment options are available in order to reduce tumor load, to inhibit tumor growth or to alleviate symptoms. Treatment options include somatostatin receptor analogs (SSA), targeted therapy, chemotherapy or peptide receptor radionuclide therapy (PRRT).

Our knowledge on pNET has improved considerably in the last decade, as is evident from the fast development of staging and grading systems proposed by the World Health Organization (WHO), the European Neuroendocrine Tumor Society (ENETS) and the American Joint Committee on Cancers (AJCC). In the present study, we provide an overview of patients diagnosed with pNET in the Netherlands identified through the nationwide Netherlands Cancer Registry (NCR). The NCR covers the complete Dutch population and receives lists of newly diagnosed cancer cases from the nationwide Automated Pathology System (PALGA) on a weekly basis [6]. In addition, lists of discharged cancer patients from the national registry of hospital discharge diagnosis are obtained to capture pNET cases with only a clinical diagnosis [7]. Checks on completeness of the data show a national coverage of about 95% [8, 9]. We aim to provide more insight into the treatment related survival of patients with pNET. This knowledge will support decisions on treatment regimens and help identify priorities in research for the future. To our knowledge, this is the first comprehensive survey on pNET epidemiology in the Netherlands.

### Patients and methods

Cases of pNET diagnosed from January 2008 to December 2013 were obtained from the nationwide, population-based NCR database, managed by the Netherlands Comprehensive Cancer Organization (IKNL). Registration and coding in this registry was conducted according to the guideline of the WHO and the International Association of Cancer Registries [10]. Topography and histology were coded according to the International Classification of Diseases for Oncology, third edition (ICD-O-3) [11]. To identify patients with neuroendocrine tumors of the pancreas ICD-O-3 codes (C251, C252, C254, C258 and C259) were combined with histology codes (8000-8011, 8013, 8041-8044, 8150-8153, 8155-8157, 8240-8242, 8246-8249, 8574 and 9990) from the PALGA network. Clinical and pathological information was obtained from hospital records.

Only patients with pancreatic NET were included. NET of other origin, as well as patients diagnosed from postmortem autopsies and tumors with mixed histology, such as adenocarcinoma of the pancreas, was excluded. Tumor–node–metastasis (TNM) assessment was based on the TNM classification 6th edition proposed by ENETS [12]. Missing TNM stage was assessed using supplementary data on “extend of disease” present in the NCR database. In addition, unrecorded data on TNM classification, tumor size and resection margins for surgically resected tumors were requested from all pathology centers and manually complemented for each patient. Data on functionality of the tumors were not present in the registry. Tumors were considered localized when the malignant tissue was confined to the pancreas, regional if there was extension into adjacent organs or metastasis to regional lymph nodes and distant if metastasis to other organs was present. Grading was performed using the WHO grading system from the time of diagnosis, meaning that patients diagnosed before 2010 were graded according to the WHO 2004 grading system, and patients diagnosed in 2010 and later using the WHO 2010 grading system. First-line treatment of all patients was recorded. Surgery was defined as surgical resection of the primary tumor. Patients who underwent resection of distant metastases were excluded from this category, as well as patients who underwent bypass surgery or an endoscopic procedure without resection of the tumor. Targeted therapy included either treatment with a tyrosine/kinase inhibitor (-nib) or everolimus. Other treatments included peptide receptor radionuclide therapy (PRRT), chemotherapy or somatostatin analogs (SSA).

Statistical analyses were performed using SPSS statistics for Windows version 23.0 (IBM Corp. Armonk, NY). On the basis of the distribution, data were described as median with interquartile range (IQR) for skewed distributions and as mean with standard deviation (SD) for normal distribution. For categorical data, the number and proportion (%) were displayed. Differences between patient groups based on tumor characteristics were investigated using a Chi-square statistic. Kaplan–Meier curves were plotted and log-rank statistics computed to detect differences between survival curves for various subpopulations. Survival was defined as the time from diagnosis until death (if known) or last follow-up (last known alive date, or December 31, 2013). Median survival was defined as the length of time, from the date of diagnosis, that half of the patients are still alive. Univariable and multivariate Cox proportional hazards regression models were used to estimate hazard ratios (HR) with 95% confidence intervals (95% CI) for factors associated with survival.

## Results

### Demographics

Patient, tumor and treatment characteristics are presented in Table 1. In total, 611 patients diagnosed with pNET were included in the analyses. The diagnosis pNET was made at an academic hospital in 36% and in a peripheral hospital in 63% of cases. Treatment was received more often in an academic hospital (46% vs. 30% peripheral hospital). Median follow-up was 25.7 months (IQR 10–45 months); all-cause mortality was 42%. Patients diagnosed with distant metastases were 53% in 2008 and 44% in 2013 (p = 0.390). Most patients had a grade 1 tumor (32%). Nodal metastases were seen in 23% of G1 tumors, 43% of G2 tumors and 71% of G3 tumors, respectively (*p* < 0.001). Distant metastases were present in 25% of G1, 51% of G2 and 71% in G3 tumors (*p* < 0.001). Nodal or distant metastases were significantly more frequent in patients with higher tumor stage (both *p* < 0.001). Patients with positive lymph nodes also had distant metastases in 62%, whereas node-negative patients had distant metastases in 27% (*p* < 0.001).

**Table 1:** Patient, tumor and treatment characteristics

	Data available *N* (%)	Overall	Surgery	PRRT	Chemotherapy	Targeted therapy	SSA	No therapy
*N*		611	255 (42%)	41 (7%)	44 (7%)	21 (3%)	62 (10%)	150 (25%)
Year of diagnosis	611 (100%)							
2008		64 (11%)	17/64 (27%)	10/64 (16%)	7/64 (11%)	0 (0%)	7/64 (11%)	16 (25%)
2009		90 (15%)	44/90 (49%)	7/90 (8%)	8/90 (9%)	2/90 (2%)	7/90 (7%)	16/90 (18%)
2010		97 (16%)	32/97 (33%)	9/97 (9%)	8/97 (8%)	2/97 (2%)	8/97 (8%)	36/97 (37%)
2011		105 (17%)	51/105 (49%)	5/105 (5%)	7/105 (7%)	3/105 (3%)	12/105 (11%)	12/105 (11%)
2012		135 (22%)	59/135 (44%)	4/134 (3%)	8/120 (6%)	8/135 (6%)	14/135 (10%)	35/135 (26%)
2013		120 (20%)	52/120 (43%)	6/120 (5%)	6/120 (5%)	6/120 (5%)	14/120 (12%)	24/120 (21%)
Median age (range)	611 (100%)	62 (53–71)	59 (19–81)	57 (38–85)	60 (38–81)	60 (63–82)	67.5 (40–87)	69 (20–90)
Gender	611 (100%)							
Male		314 (51%)	121 (48%)	20 (49%)	26 (59%)	8 (38%)	40 (65%)	74 (49%)
Female		297 (49%)	134 (53%)	21 (51%)	18 (41%)	13 (62%)	22 (36%)	76 (51%)
Tumor grade	348 (57%)							
G1		197 (32%)	143 (56%)	6 (15%)	3 (7%)	2 (10%)	18 (29%)	20 (13%)
G2		101 (17%)	56 (22%)	7 (17%)	2 (5%)	8 (38%)	16 (26%)	8 (5%)
G3		50 (8%)	12 (5%)	1 (2%)	11 (25%)	3 (14%)	0 (0%)	19 (13%)
T-stadium	462 (76%)							
T1		131 (22%)	99 (39%)	0 (0%)	1 (2%)	1 (5%)	1 (2%)	28 (19%)
T2		172 (28%)	82 (32%)	12 (29%)	10 (23%)	6 (29%)	17 (27%)	38 (25%)
T3		117 (19%)	62 (24%)	6 (15%)	8 (18%)	8 (38%)	10 (16%)	16 (11%)
T4		42 (7%)	3 (1%)	4 (10%)	6 (14%)	2 (10%)	13 (21%)	10 (7%)
N-stadium	479 (81%)							
N0		315 (52%)	181 (71%)	14 (34%)	16 (36%)	11 (52%)	22 (36%)	62 (41%)
N+		182 (30%)	69 (27%)	10 (24%)	15 (34%)	6 (29%)	18 (29%)	47 (43%)
M-stadium	246 (96%)							
M0		314 (51%)	232 (91%)	9 (22%)	3 (7%)	1 (5%)	7 (11%)	50 (33%)
M+		290 (48%)	20 (8%)	31 (76%)	40 (91%)	20 (95%)	55 (89%)	98 (65%)
Deaths	611 (100%)	259 (42%)	35 (14%)	20 (49%)	39 (89%)	12 (57%)	33 (53%)	98 (65%)
5-year survival		53%	86%	33%	21%	Not reached	24%	30%
Median survival		25.7 months	36.2 months	43.6 months	7.6 months	16.2 months	23.1 months	9.9 months

### Survival

Overall, 5-year survival was 53%. There was no significant difference in overall survival for patients diagnosed in different years separately analyzed. Five-year survival was 78% without and 27% with distant metastases (*p* < 0.001). In the absence of lymph node metastases, 5-year survival was 72%, compared to 44% in patients with nodal metastases (*p* < 0.001). In the absence of distant metastases, positive lymph nodes had a significant negative effect on survival (*p* = 0.003). With distant metastases, the effect of lymph node metastases on survival was not significant, however close (*p* = 0.053).

**Fig. 1:**
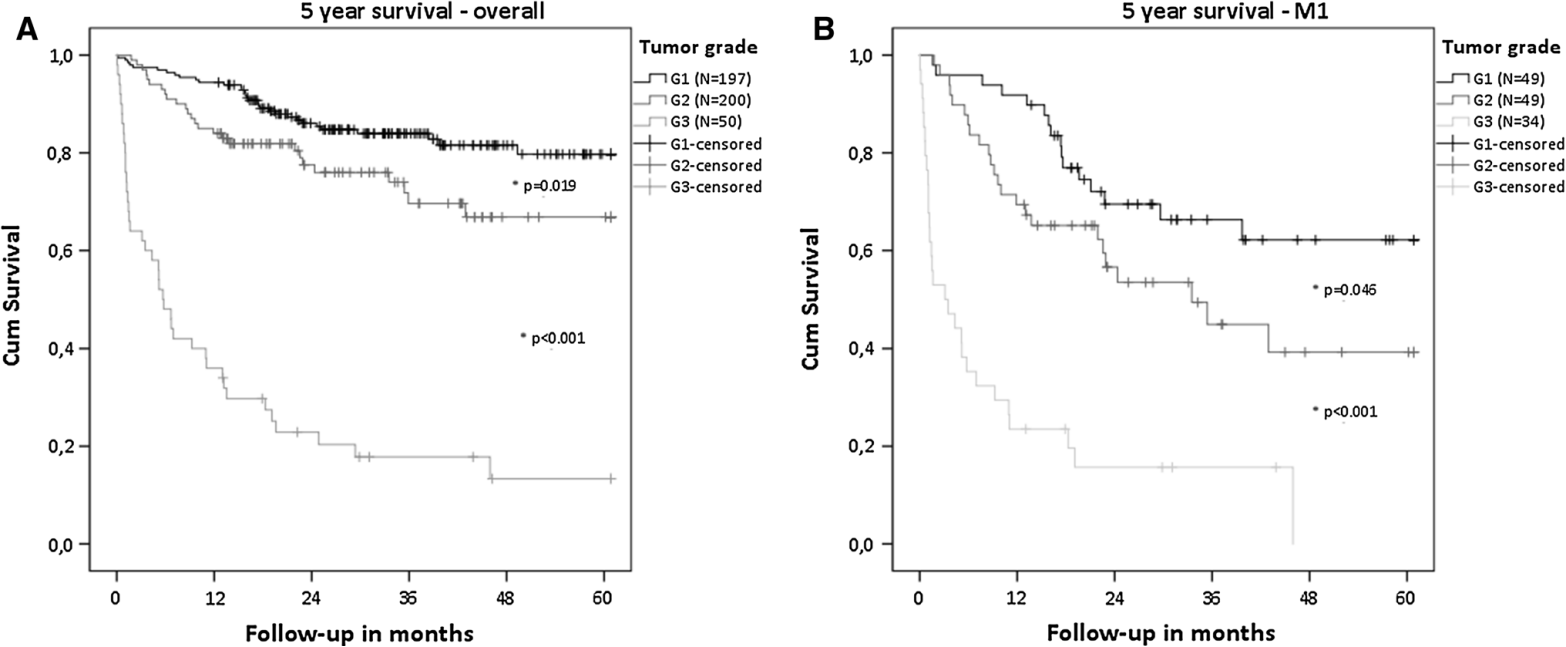
Overall survival of patients with different tumor grades. **a** Overall patient population. **b** Patient with metastatic disease

A higher tumor grade was associated with worse survival, in both localized as well as distant metastatic disease (Fig. 1). Overall, 5-year survival was 80% for G1, 67% for G2 and 13% for G3 tumors. Median survival was decreased by 7.4 months for G1 (*p* < 0.001), 11.3 months for grade 2 (*p* < 0.001) and 12.4 months for grade 3 tumors (*p* = 0.022) in the presence of distant metastases. Nodal metastases (N0 vs. N1) were not associated with a survival difference of patient with different tumor grade.

**Fig. 2:**
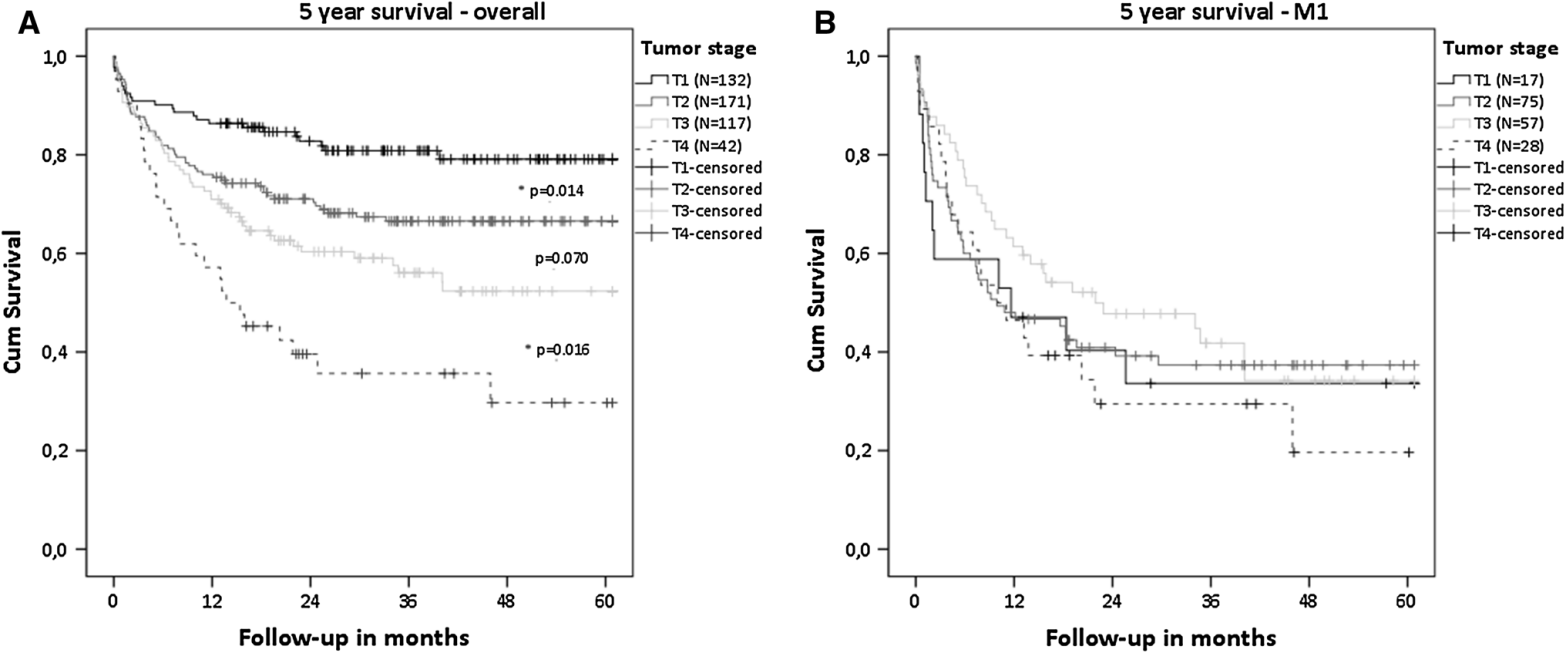
Overall survival of patients with different tumor stages. **a** Overall patient population. **b** Patients with metastatic disease. No significant difference in survival was seen between each tumor stage

Survival was worse with higher tumor stage in patients with localized disease (Fig. 2a). Five-year survival was 79% for T1, 67% for T2, 52% for T3 and 30% for T4 tumors. In the presence of distant metastases, increase in tumor stage showed no worsening of survival (Fig. 2b). Median survival with and without distant metastases was 33.1 versus 10.1 months for T1 tumors (*p* < 0.001), 36.3 versus 9.2 months for T2 tumors (*p* < 0.001) and 25 versus 16.6 months for T3 tumors (*p* = 0.002), respectively. In T4 tumors, M0 and M1 patients had comparable survival curves (*p* = 0.074).

### Surgical and other treatment

Resection of the primary tumor was performed in 255 cases (25%). The number of patients who underwent surgery increased from 27% in 2008 to 43% in 2013 (*p* = 0.02). Chemotherapy, PRRT, targeted therapy or SSA was received by 168 patients. Hundred and fifty patients received no treatment. Patient and tumor characteristics per treatments are presented in Table 1. Tumor size was only known for patients who underwent surgical resection. Lymph node metastases were detected in 16% of tumors < 2 cm, 38% of tumors of 2–4 cm and 37% of tumors > 4 cm in size (*p* = 0.002).

**Fig. 3:**
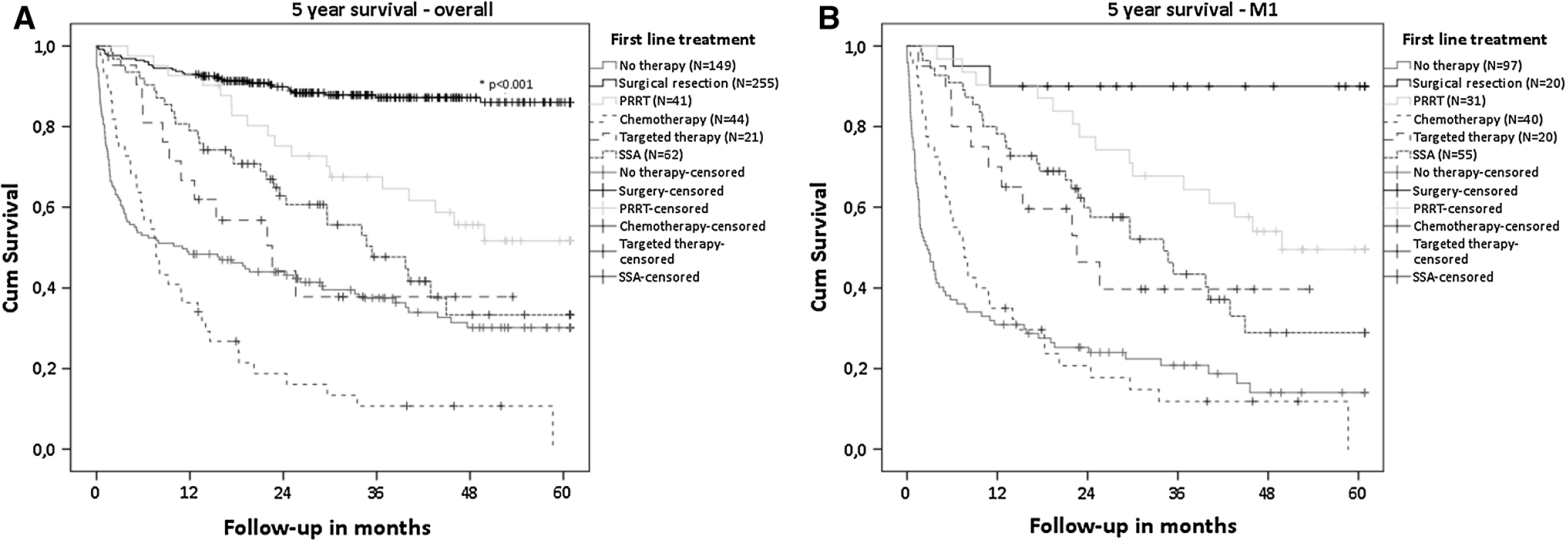
Overall survival of patients based on first-line treatment **a** all patients. Surgical resection showed significantly superior survival compared to the other treatments (all *p* < 0.001). **b** Patients with distant metastases

**Fig. 4:**
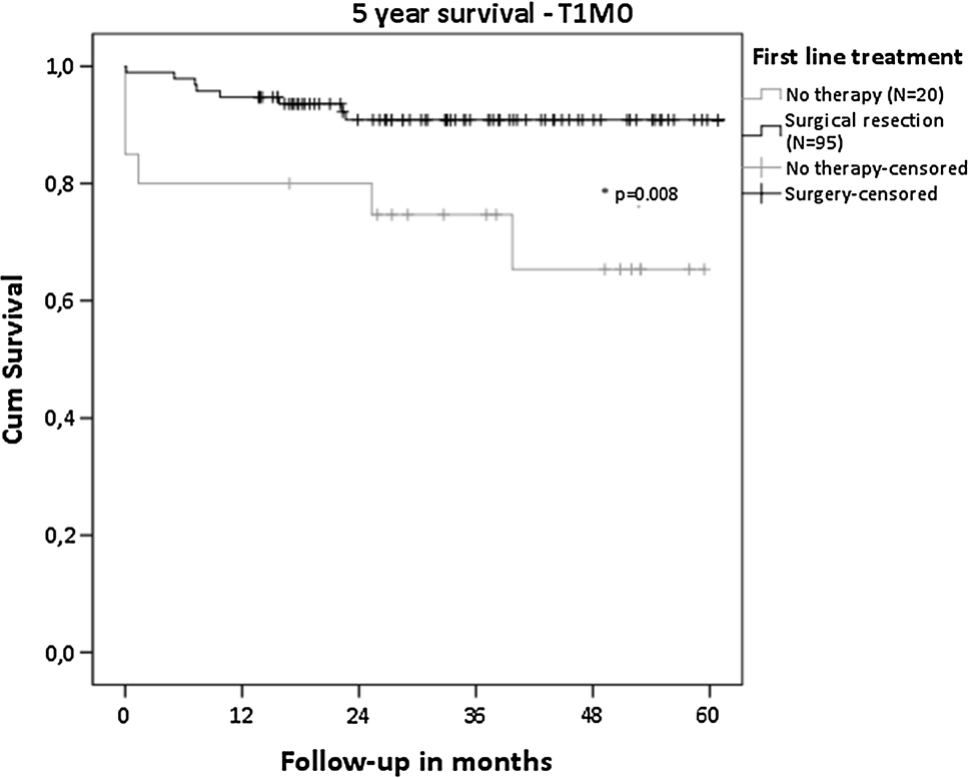
Survival of patients with T1M0 tumors

Overall, survival was favorable for patients who underwent surgical resection of the primary tumor compared to PRRT, chemotherapy, targeted therapy, SSA and no therapy (all *p* < 0.001, Fig.  3a). For patients with the most indolent tumors (T1M0), a significant survival benefit was seen for surgical resection compared to no treatment (*p* = 0.008), with a 5-year survival of 91 versus 65% (Fig. 4—T1M0). Focusing on patients with distant metastatic disease, surgical resection of the primary tumor showed a significant better survival, with a 5-year survival of 90% compared to 50% for PRRT (*p* = 0.016), 29% for SSA (*p* < 0.001) and 14% for no therapy (*p* < 0.001)(Fig. 3b). Five-year survival of patients receiving chemotherapy (*p* < 0.001) or targeted therapy (*p* = 0.002) was not reached. When surgical resection was not performed in the presence of distant metastases, patients who received PRRT showed significant better survival compared to chemotherapy (*p* < 0.001) or SSA (*p* = 0.04) but not to targeted therapy (*p* = 0.062). Tumor grade significantly differed in this population between patients who received PRRT and chemotherapy (*p* = 0.002) and between chemotherapy and targeted therapy (*p* = 0.017). Other patient and tumor characteristics were not significantly different between the treatment groups.

### Predictors for survival

Age at diagnosis, tumor grade, tumor stage, lymph node metastases, distant metastasis and first-line treatment showed an association with survival in univariable analysis. Multivariable analysis showed that G3 tumors (HR 7.23, 95% CI 3.25–16.13) and surgical resection (HR 0.12, 95% CI 0.05–0.30) were independently associated with survival (Table 2). Excluding G3 tumors from multivariable analysis resulted in comparable results for surgical resection (HR 0.12, 95% CI 0.04–0.38) and only an additional significance for age without clinical relevance (HR 1.03, 95% CI 1.00–1.07).

**Table 2:** Cox regression analysis

Risk factors	Univariable			Multivariable		
	HRs	95% CI	*p* value	HRs	95% CI	*p* value
Gender	1.03	0.81–1.31	0.817	–	–	–
Age at diagnosis	1.04	1.03–4.05	< 0.001	1.03	1.00–1.05	0.061
Year of diagnosis						
2008	Ref.	Ref.	Ref.	–	–	–
2009	0.92	0.60–1.43	0.73			
2010	0.95	0.61–1.48	0.81			
2011	0.67	0.42–1.07	0.09			
2012	0.93	0.60–1.44	0.74			
2013	0.93	0.58–1.50	0.77			
Tumor grade						
Gl	Ref.	Ref.	Ref.	Ref.	Ref.	Ref.
G2	1.87	1.13–3.10	0.015	1.43	0.67–3.09	0.358
G3	11.1	7.0–17.57	< 0.001	7.94	3.61–17.48	< 0.001
Tumor stage						
Tl	Ref.	Ref.	Ref.	Ref.	Ref.	Ref.
T2	1.75	1.11–2.77	0.016	0.66	0.29–1.50	0.317
T3	2.39	1.50–3.82	< 0.001	0.96	0.42–2.19	0.925
T4	4.25	2.49–7.26	< 0.001	0.79	0.26–2.39	0.678
Nodal status	2.62	1.95–3.54	< 0.001	1.22	0.70–2.12	0.492
Distant metastasis	4.79	3.60–6.37	< 0.001	1.31	0.59–2.89	0.504
First-line treatment						
No therapy	Ref.	Ref.	Ref.	Ref.	Ref.	Ref.
Surgical resection	0.12	0.08–0.18	< 0.001	0.13	0.05–0.31	< 0.001
Chemotherapy	1.63	1.12–2.37	0.011	0.50	0.20–1.16	0.104
Other systemic therapy	0.62	0.44–0.88	0.008	0.83	0.32–2.18	0.708
Nuclear treatment	0.47	0.31–0.73	0.001	0.79	0.28–2.52	0.665

## Discussion

In this study, we present the treatment and survival of patients diagnosed with pNET in the Netherlands over a 6-year period. Patients undergoing surgical resection show superior outcomes in terms of survival, regardless of the presence of distant metastases. Apart from surgery and allowing for selection bias, PRRT shows the best survival curves in patients with disseminated disease.

For a long time, surgical treatment was the gold standard for patients diagnosed with localized pNET. However, there have recently been changes in the guidelines advising a conservative, rather than a surgical approach, for small non-functional tumors [13]. Data supporting this observational option are controversial as is evident from the presented results: T1M0 patients with a resection have a survival benefit compared to those without treatment. Still, issues of selection bias, small sample and missing data limit our ability to make valid conclusions. Similar studies support or contradict our findings, indicating comparable study bias and the need for prospective data [14–16]. It is imaginable that the reason to refrain from surgery might influence the outcome in both directions. As there are no RCTs or meta-analyses that can assist the optimal management of small pNET, a prospective study to register and monitor all patients with small pNET (the PANDORA study) is currently being conducted in the Netherlands.

A more aggressive approach has increasingly been described in the literature with regards to metastasized disease [17–20]. Similarly, our results promote surgical resection for patients with distant metastases, with a survival benefit of 40–76% in 5 years. Inclusion bias, with relative stable M1 patients, warrants that future studies clearly describe patients-related treatment determinants, tumor progression and time to progression as markers. Definitions of metachronous and synchronous metastases should be established, preferably in international guidelines, for research to be univocal and comparable. Only then, the presented results can be confirmed in prospective trials that weigh the effect of resection in the presence of metastases (i.e., resection of the primary tumor with/without synchronous resection of solitary liver metastases) against the current, less invasive, systemic and nuclear options, taking into account the risks of both treatments.

The effect of PRRT has not previously been described in a population-based study. In this cohort, 41 patients received treatment with PRRT and showed the longest median survival compared to other treatments. Furthermore, survival analyses showed that PRRT had comparable outcomes to surgical resection in the overall population, and favorable outcome in patients with distant metastatic disease who did not undergo surgery. Nevertheless, there is a clear selection bias since less G3 tumors received PRRT compared to chemotherapy, implying that the favorable outcome of PRRT might be explained by the selection of patients with less aggressive disease. Significant differences for tumor grade between the treatment groups support this theory. Unfortunately, the available data were too small to provide reliable subanalyses on tumor grade for the non-surgical treatment groups.

The results of this study must be seen in light of its limitations. Data were evaluated retrospectively, and pathology reports were not standardized at the time of treatment. This may explain the considerable amount of missing data for tumor stage and grade, as other population-based studies also report [1, 21]. It is worth mentioning that registration improved up to 78% for grade and 89% for tumor stage in 2013. An additional increase is anticipated in the Netherlands as national pathological guidelines for pNET have been published the in 2013, and 4 hospitals have been named ENETS Centers of Excellence after the study period. Nevertheless, the amount of patients treated in non-academic centers show that there may be bias due to lack of centralization, as pNET requires complex knowledge and care. Furthermore, heterogeneity remains a difficult and recurring issue in pNET research. Accurate assessment of patient and tumor characteristics, along with strict selection criteria in future studies, should be pursued to limit bias and draw reliable conclusions from study results.

## Conclusion and future perspectives

Despite efforts, the overall survival of patients diagnosed with pNET is not improving. An effective and purposeful treatment approach is therefore necessary. Besides survival, patient-related outcomes should be included in future studies.

Tumor grade and TNM stage remain the most important prognostic factors and need to be clearly defined in each patient, to determine prognosis and treatment. Surgical treatment of small pNET and patients with M1 disease improves survival compared to all other treatments. Prospective trials must be encouraged to achieve fast and reliable results. Emphasis of future research should be on whether or not to resect pNET in patients with small lesions as well as patients with distant metastatic disease. Clear definitions for synchronous/metachronous lymph node and distant metastases, time to progression and treatment indication should be established and used in all studies concerning pNET.

## References

[1] Yao JC, Hassan M, Phan A (2008). One hundred years after “carcinoid”: epidemiology of and prognostic factors for neuroendocrine tumors in 35,825 cases in the United States. J Clin Oncol.

[2] Modlin IM, Oberg K, Chung DC (2008). Gastroenteropancreatic neuroendocrine tumours. Lancet Oncol.

[3] Modlin IM, Lye KD (2003). Kidd M A 5-decade analysis of 13,715 carcinoid tumors. Cancer.

[4] Metz DC, Jensen RT (2008). Gastrointestinal neuroendocrine tumors: pancreatic endocrine tumors. Gastroenterology.

[5] Kloppel G (2007). Tumour biology and histopathology of neuroendocrine tumours. Best Pract Res Clin Endocrinol Metab.

[6] Casparie M, Tiebosch AT, Burger G (2007). Pathology databanking and biobanking in The Netherlands, a central role for PALGA, the nationwide histopathology and cytopathology data network and archive. Cell Oncol.

[7] van der Zwan JM, van Dijk BA, Visser O (2015). Rare cancers in The Netherlands: a population-based study. Eur J Cancer Prev.

[8] Goldbohm R, van der Brandt P, Dorant E (1994). Estimation of the coverage of Dutch municipalities by cancer registries and PALGA based on hospital discharge. TSG.

[9] Schouten LJ, Hoppener P, van den Brandt PA (1993). Completeness of cancer registration in Limburg, The Netherlands. Int J Epidemiol.

[10] Jensen OM, Storm HH (1991). Cancer registration: principles and methods. Reporting of results. IARC Sci Publ.

[11] Fritz AG (2000). International classification of diseases for oncology: ICDO.

[12] Rindi G, Kloppel G, Alhman H (2006). TNM staging of foregut (neuro)endocrine tumors: a consensus proposal including a grading system. Virchows Arch.

[13] Falconi M, Eriksson B, Kaltsas G (2016). ENETS consensus guidelines update for the management of patients with functional pancreatic neuroendocrine tumors and non-functional pancreatic neuroendocrine tumors. Neuroendocrinology.

[14] Bar-Moshe Y, Mazeh H, Grozinsky-Glasberg S (2017). Non-functioning pancreatic neuroendocrine tumors: surgery or observation?. World J Gastrointest Endosc.

[15] Guo J, Zhao J, Bi X (2017). Should surgery be conducted for small nonfunctioning pancreatic neuroendocrine tumors: a systemic review. Oncotarget.

[16] Ricci C, Taffurelli G, Campana D (2017). Is surgery the best treatment for sporadic small (≤ 2 cm) non-functioning pancreatic neuroendocrine tumours? A single centre experience. Pancreatology.

[17] Fairweather M, Swanson R, Wang J (2017). Management of neuroendocrine tumor liver metastases: long-term outcomes and prognostic factors from a large prospective database. Ann Surg Oncol.

[18] Citterio D, Pusceddu S, Facciorusso A (2017). Primary tumour resection may improve survival in functional well-differentiated neuroendocrine tumours metastatic to the liver. Eur J Surg Oncol.

[19] Almond LM, Hodson J, Ford SJ (2017). Role of palliative resection of the primary tumour in advanced pancreatic and small intestinal neuroendocrine tumours: a systematic review and meta-analysis. Eur J Surg Oncol.

[20] Partelli S, Inama M, Rinke A (2015). Long-term outcomes of surgical management of pancreatic neuroendocrine tumors with synchronous liver metastases. Neuroendocrinology.

[21] Halfdanarson TR, Rabe KG, Rubin J (2008). Pancreatic neuroendocrine tumors (PNETs): incidence, prognosis and recent trend toward improved survival. Ann Oncol.

